# Can’t Disconnect Even After-Hours: How Work Connectivity Behavior After-Hours Affects Employees’ Thriving at Work and Family

**DOI:** 10.3389/fpsyg.2022.865776

**Published:** 2022-03-09

**Authors:** Yang Yang, Rui Yan, Yan Meng

**Affiliations:** School of Management, Harbin Institute of Technology, Harbin, China

**Keywords:** work connectivity behavior after-hours (WCBA), work–family enrichment, work–family conflict, thriving at work, thriving at family, Johnson–Neyman’s method

## Abstract

As more organizations adopt telecommuting or working from home, the work-connected behavior of their employees during non-working hours increases, weakening the boundary between work and family. However, no study has clearly identified whether and how work connectivity behavior after-hours (WCBA) affects employees’ work and family status. Therefore, using role theory, we explored the mechanisms by which WCBA affects employees’ thriving at work and family through work–family enrichment and work–family conflict, and compared the impact of different levels of support for family members on work–family enrichment and conflict, using the Johnson–Neyman method. Our analysis of two-wave data from 257 employees led to the following findings. (1) WCBA had a positive impact on thriving at work, but not on family. (2) There is a ‘double-edged sword’ effect on the impact of WCBA on thriving at work, meaning that work–family enrichment can positively influence thriving at work and negatively influence work–family conflict. (3) There is a double-edged sword effect on the impact of WCBA on thriving at family, meaning that work–family enrichment can positively influence thriving at family and negatively influence work–family conflict. (4) The support of family members moderates the double-edged sword effect between WCBA and thriving at work, in that it can strengthen the positive effects of work–family enrichment (below 3.32 points or above 4.19 points) and weaken the negative effects of work–family conflicts (below 4.28 points). (5) Support from family members reinforces the positive impact of work–family enrichment (above 3.46 points) on thriving at family. Thus our study reveals the mechanisms by which WCBA affects the thriving at work and family of employees, and identifies potential methods for managing different levels of work–family enrichment and work–family conflict from the perspective of family member support.

## Introduction

Telecommuting, which has been widely used during the COVID-19 pandemic, has not been rolled back as the COVID-19 pandemic comes under effective control. Instead, organizations and employees are actively adapting to this ‘new normal’ (Solomon, 2020). For example, Twitter, Facebook, Microsoft, and other companies are allowing their employees to work remotely on a permanent basis. However, telecommuting brings new challenges in keeping employees working properly. That is, it exacerbates the penetration of work connectivity from working to non-working hours (Mazmanian, 2013), which has a significant impact on the work and family lives of employees (Colbert et al., 2016).

Thriving, as a positive psychological state of employees, has a positive impact on employees’ work and family lives. Most relevant studies show that thriving is good for organizational performance and employee wellbeing (Paterson et al., 2014), and is fundamental for promoting sustainable work-life development (Spreitzer et al., 2012). These studies mostly focus on thriving at work, providing rich information in terms of antecedent variables, outcome variables, influence mechanisms, and boundary conditions (Walumbwa et al., 2018). However, studies have paid insufficient attention to prosperity in the non-work field, especially in the family field, which is closely related to employees. Porath et al. (2012) point out that thriving at work and thriving at family are two different variables, and that maintaining both kinds of prosperity at the same time is challenging. However, some studies find that in the case of abundant resources for employee work, prosperity may expand into the family sphere (Carmeli and Russo, 2016), and increased autonomy also promotes thriving at family (Chen, 2020). In a telecommuting scenario, while work connectivity after-hours can provide role-integrated resources for work prosperity, it can also lead to competition for family roles and resources, which has an impact on thriving at family. Therefore, it is important to explore how non-working-time connectivity affects both employees’ work and their family prosperity.

Research shows that handling work tasks during non-working hours blurs the boundaries between an employee’s role at work and at home (Kossek et al., 2006), but there is no consensus on its impact. Work connectivity after-hours can provide autonomy for the integration and transformation of employee roles, which helps to form role identity and thriving at work (Rau and Hyland, 2002). It can also guide the positive flow of work resources to the family field (Ragsdale and Hoover, 2016), which helps to achieve work and family prosperity. However, the expectation for a continuous connection between colleagues and leaders may not only undermine the autonomy of employee role integration and transformation (Fujimoto et al., 2016)—leading to negative emotions such as work anxiety and burnout, and inhibiting thriving at work (Ashforth et al., 2000)—it can also interrupt employees’ non-work activities (Cavazotte et al., 2014), which destroys the harmony between work and family. Therefore, exploring the ‘double-edged sword’ effect of work connectivity after-hours on work and family is of great significance for a deeper understanding of how non-work-time connectivity affects work and family prosperity.

While deepening the integration of work and family, work connectivity behavior after-hours (WCBA) highlights the influence of family members on the role transformation of employees, and its support can help employees better meet their role expectations from work and family spheres (Adkins and Premeaux, 2014). Stronger family member support only provides instrumental and emotional support, which can help employees better meet their job role expectations, but also reduces expectations of their family roles, which can help to alleviate the difficulty of role conversion faced by employees, and promote positive work attitudes such as work–family enrichment and employee prosperity (Bhave and Lefter, 2018). Conversely, weaker family member support may increase expectations of employees’ family roles; reduce support for, and hinder them in, performing their work roles; result in employees facing greater role pressure and difficulty in the role conversion process; and promote negative behavior such as anti-production behavior, and work and family withdrawal (Menges et al., 2017). Therefore, exploring the boundary effects of family member support will help deepen understanding of when and how WCBA affects employee and family prosperity through work–family enrichment and conflict.

**FIGURE 1:**
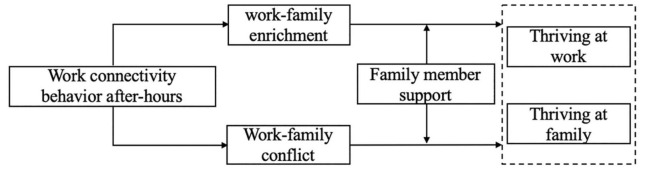
The theoretical models.

In summary, with reference to role theory, we analyzed the double-edged sword effect between WCBA and thriving of employees and families (Figure 1). Further, we used the Johnson–Neyman (J–N) method to explore the boundary effect of family member support on this double-edged sword effect, which provides a reference for enterprises to maintain work–family balance and promote the thriving of employees in both their work and family roles during non-working hours. In the following sections: First, we briefly construct the conceptual framework through review of the literature and develop the hypotheses. Second, we describe the sampling and data collection procedure, measurement items. Third, we present the test results and finding. Finally, we discuss the results, theoretical and practical implications of research, concludes, limitation and future research directions.

## Theoretical Background and Hypotheses

### Work Connectivity Behavior After-Hours and Individual Thriving

Work connectivity behavior after-hours refers to the behavior of employees who use communication technology to contact colleagues to participate in work during non-working hours (Richardson and Benbunan-Fich, 2011). This behavior helps establish a connection between the workplace and the non-workplace (Davis, 2010), gives employees greater freedom to enhance their sense of job control and their ability to integrate roles (Mazmanian, 2013), provides employees with more work resources to cope with pressures from work and life, and creates the possibility for employees to handle multiple roles and perform their responsibilities at the same time (Kreiner, 2006). This behavior allows employees to work in different places and times (O’Leary and Cummings, 2007)—which can help them to take on multiple roles at work, such as representing the organization in interactions with suppliers and acting as subordinates in communication with leaders (Reinsch et al., 2008)—and also provides a way for employees to use commuting time and non-working hours to carrying out their responsibilities (Dery and Maccormick, 2012), such as taking on family roles. Further, WCBA not only helps improve the efficiency of work interaction (Edmondson, 1999), communication, and exploration between employees and colleagues, but also helps reduce the impact of meeting pressure, communication pressure, and a negative organizational climate faced by employees (Fonner and Roloff, 2010), providing more work resources for promoting employee learning and growth (Carmeli et al., 2009).

Thriving refers to a dynamic state of mind in which employees are able to experience both ‘vitality’ and ‘learning’ at the same time. Thriving at work refers to the feeling of being energetic at work and being able to continuously learn new knowledge and skills (Spreitzer et al., 2005), while thriving at family refers to the feeling of being energetic in the family and being able to continuously learn new knowledge and skills (Carmeli and Russo, 2016). Thriving, as a subjective experience, promotes not only individual growth, but also positive behavior in work and life (Bugental, 2004), such as showing better performance (Paterson et al., 2014), organizational citizenship behavior (Porath et al., 2012), innovative behavior, and career growth at work (Wallace et al., 2013), and showing more helpful behavior, self-development, and better fulfillment of non-work roles in life (Spreitzer et al., 2005). The cited studies are based on the social embedding model proposed by Spreitzer et al. (2005), from the perspective of obtaining relationship resources (Guan and Frenkel, 2020) and leadership support at work (Rego et al., 2020), to examine how to promote thriving at work. On the other hand, based the social embedding model, research on the thriving at work of employees and the thriving at families is carried out from the perspective of non-work care (Carmeli and Russo, 2016).

Although many studies have shown that leadership behavior, intra-organizational relationship resources, and employee out-of-organization communication have an important effect on individual thriving, the influence of scenario factors such as the level of autonomous decision making and degree of organizational information sharing on individual thriving is still in a “black box” (Spreitzer and Porath, 2013; Carmeli and Russo, 2016). As a new work scenario, non-working-time work connectivity can stimulate and create learning opportunities and promote employees’ personal development; give them greater autonomy in decision making; and accelerate organizational information sharing (Bandura, 1988; Spreitzer et al., 2012). This act of connectivity creates opportunities for employees to learn and improve by creating a safe environment for communication and work, enhancing their ability to access formal and informal information and resources from the organization’s social network. At the same time, it helps protect the privacy of employee performance feedback, build a bridge between supervisors and subordinates, and promote the development of employees in the organization. Further, this act of connectivity enables employees to apply learning at work to the home field, realizing the possibility of assuming multiple roles simultaneously to maintain boundaries between work and family, creating opportunities for employees to learn and improve in the family field, and enabling them to thrive in the family (Kirby et al., 2012), for example: the communication skills learned on the job can be applied to family communication. Thus, we propose:

Hypotheses: Work connectivity behavior after-hours will positively influence thriving at work (H1a) and family (H1b).

### The Mediating Role of Work–Family Enrichment

Work–family enrichment refers to the process in which employees share resources obtained in the work field to the family field in the mutual penetration of work and family, and achieve work–family enrichment (Powell and Greenhaus, 2006; Lapierre et al., 2018). Employees experience role inertia in the transition between work and family roles, which helps them use the skills gained in the work role to take on family roles, achieving work-to-family gains (Fujimoto et al., 2016). Studies have shown that work–family enrichment not only alleviates work and family pressures faced by employees, but facilitate the flow of work resources such as work skills, social capital, and flexible work arrangements to the family field (Carlson et al., 2011, 2019), improving work and family satisfaction (Tang et al., 2014). Although these studies provide rich information on the antecedents and consequences of work–family enrichment in terms of work resources, little attention has been paid to the extension of work scenarios, and only a small number of scholars has explored the impact of flexible work arrangements and other work scenarios on work–family enrichment and family satisfaction (Carlson et al., 2019).

Work connectivity behavior after-hours gives employees more autonomy and control, makes it possible for them to integrate multiple different role tasks, and helps them balance the needs of work and family (Boswell and Olson-Buchanan, 2007). WCBA not only helps to reduce the information exchange and meeting pressure perceived by employees (Fonner and Roloff, 2010), improving their communication efficiency, but also helps them to quickly respond to the work needs of organizations and colleagues to enhance their own status, obtaining more work resources for work–family enrichment and positive work behavior (Taheri et al., 2020), which is conducive to the generation of thriving at work (Luthans and Youssef, 2004; Bloom et al., 2011). Also, WCBA can enhance an employee’s sense of autonomy and control over role switching, alleviate the role conflict between taking on work tasks and caring for the family, help to improve the employee’s work–family balance, and promote thriving at family (Chou and Cheung, 2013). Thus, we propose:

Hypotheses: Work–family enrichment will mediate the positive influence of work connectivity behavior after-hours on thriving at work (H2a) and family (H2b).

### The Mediating Role of Work–Family Conflict

Conflict between work and family is a result of employees’ inability to meet the needs of their roles in both the work and family field (Greenhaus and Beutell, 1985). Work and family are two important areas of employees’ lives; thus, conflict between work and family will not only increase psychological pressure on employees, but also reduce their prosperity (Schieman and Glavin, 2011). To alleviate the impact of work–family conflict on employees’ psychology and work, scholars have conducted research on work disengagement (Boswell and Olson-Buchanan, 2007), organizational climate, leadership (Munir et al., 2012), and organizational and family support (Kalliath et al., 2015), achieving remarkable results. However, most studies have paid insufficient attention to changes in work scenarios. As one of the main work scenarios for employees, WCBA can enhance employees’ sense of work control to improve the fit between work and family (Rau and Hyland, 2002; Kelly et al., 2014). However, continuous connection may also make employees unable to leave the workplace in time, resulting in them not being able to respond to family expectations and assume family roles in a timely manner (Kossek et al., 2011).

Work connectivity behavior after-hours provides employees with more flexible work arrangements, enhances their sense of work control, and helps them improve work efficiency; however, it also disrupts standard work plans, resulting in irregular dynamic working hours and continuous work connections (Su and Dunifon, 2017), and weakening the boundary between work and family. This is the main cause of work–family conflict (Igbaria and Guimaraes, 1999). Further, this act of connectivity makes it difficult for employees to distinguish between work and home spheres, and they may face contradictions between colleagues’ expectations of connection response and family members’ expectations of family roles (Richardson and Benbunan-Fich, 2011). This overlapping role conflict is likely to create work–family conflict (Butts et al., 2013). Also, this act of connectivity interrupts the continuity of employee roles (Sonnentag et al., 2018)—not only their family role, but also their work roles (Barley et al., 2010), resulting in the paradox of employee autonomy (Putnam, 2014). Eventually, an employee’s sense of job control and autonomy in the process of connection will decline (Stephens et al., 2013), and they will neither be able to perform better in their family roles, nor better respond to the expectations of colleagues, resulting in the loss of thriving at work and family. Thus, we propose:

Hypotheses: Work–family conflict will mediate the negative influence of work connectivity behavior after-hours on thriving at work (H3a) and family (H3b).

### The Moderating Role of Family Member Support

Family members, as maintainers of the work–family boundary, have high sensitivity to border penetration, which affects the work behavior of employees (Maccormick et al., 2012), such as reducing the turnover intention, increasing work engagement, and improving job satisfaction. The support of family members refers to reducing family pressure faced by employees in performing their work during non-working hours, by sharing the family role that employees need to assume and reducing expectations of employees to perform their family role (Clark, 2000). Support from family members helps employees put more energy and resources into their work and enhances their thriving at work (Menges et al., 2017). At the same time, employees are more willing to extend the wealth of resources accumulated at work to the family, and take on more family roles to give back to family members to support and promote thriving at family. It can be concluded that when employees perceive stronger family member support, the positive impact of work–family enrichment on thriving at work and family might be enhanced. Conversely, the positive impact of work–family enrichment might be reduced. Thus, we propose:

Hypotheses: Family member support will moderate the influence of work–family enrichment on thriving at work (H4a) and family (H4b), such that this influence will be more positive when the employee has a high level of family member support and less positive with a low level of family member support.

The support of family members can assist employees in terms of flexible role switching, rapid immersion into different roles, and role complementarity, helping to reinforce the positive effects of WCBA. Through the analysis of the mediating role of work–family enrichment and the moderating role of family member support, it can be seen that family member support is an important boundary condition for work–family enrichment to have a positive impact on the relationship between WCBA and thriving at work and at family. Thus, we propose:

Hypotheses: Family member support will moderate the mediating role of work–family enrichment in the relationship between work connectivity behavior after-hours and thriving at work (H5a) and family (H5b), such that the mediating effect will be more positive with a high level of family member support and less positive when family member support is at a low level.

To maintain the orderly conduct of family activities, family members increase expectations of employees’ family roles to maintain the boundaries between work and family. Since WCBA can give employees more autonomy, family members might increase expectations of employees in fulfilling their family roles, such as taking on more chores and caring for children (Adkins and Premeaux, 2014). However, a high level of family member support will reduce expectations of employees’ family roles, alleviate conflict between work and family roles, help employees better respond to work expectations from organizations and colleagues, and promote thriving at work (Presti et al., 2016). At the same time, a reduction in family disturbance can help to reduce work–family conflict and promote the continuation of thriving from work to family (Wu et al., 2010). When employees perceive stronger family member support, the negative impact of work–family conflict on thriving at work and family will be weakened. Conversely, the negative effects of work–family conflicts will be reinforced. Thus, we propose:

Hypotheses: Family member support will moderate the influence of work–family conflict on thriving at work (H4c) and family (H4d), such that this influence will be less negative when an employee has strong family member support and more negative when there is a low level of family member support.

Family member support can also reduce role conflict and ambiguities for employees in role transition, enhance the matching of employees in role transition with work and family scenarios, and help to alleviate the negative impact of WCBA. Through the analysis of the mediating role of work–family conflict and the moderating role of family member support, it can be seen that family member support is an important boundary condition that affects the negative impact of work–family conflict on the relationship between WCBA and thriving at work and family. Thus, we propose:

Hypotheses: Family member support will moderate the mediating role of work–family conflict in the relationship between work connectivity behavior after-hours and thriving at work (H5c) and family (H5d), such that the mediating effect will be less negative with a high level of family member support and more negative with a low level of family member support.

## Materials and Methods

### Participants and Procedure

To test our conceptual model, we recruited 376 participants through the online platform Credamo in China. To avoid the influence of common method bias on the conclusions of our study, we collected our data at two different times. In the first wave, after removing samples with incomplete information, we retained 300 valid samples and paid each participant US$0.47. This wave included core variables such as WCBA, WFE, TW, and control variables such as gender, age, position and education. Two weeks later, we retained 257 valid samples and also paid US$0.47 to each participant in this second wave (Gong et al., 2020). This wave included core variables such as WFC, TF, WS, and control variables such as marital status, child, parents help and home work space. We ultimately employed 257 valid samples (i.e., a response rate of 79.8%) for our analysis.

Among the valid participants, 53% were female and 47% were male. In regard to age, 49% were aged 18–30 years, 44% were 31–40, 6.6% were 41–50, and 0.4% were 51–60. For education, 1.2% were graduated from high school and below, 7.7% held a 3-year college diploma, 80.2% held a bachelor degree, and 10.9% had a master degree. In terms of their work position, 33% were ordinary employees, 41% were front-line managers, 25% were middle managers, and 1% were senior managers. Regarding marital status, 12.8% were unmarried, and 87.2% were married. Fourteen % had no children, 72% had one child, and 14% had more than one child. A 1% had no help from their parents, 3% rarely had help, 40% occasionally, and 56% often had help. Regarding the work environment at home, 49% had an enclosed independent office space, 33% had a non-enclosed independent office space, and 18% had an open office space.

### Measures

On the basis of our research goals, we measured the core variables using a previously validated scale. As all survey items were originally developed in English, we invited two scholars in organizational behavior to translate them into Chinese and then back into English following the commonly used back translation procedure, and respondents were invited to rate statements from strongly disagree to strongly agree, using scores of 1–5 on a five-point Likert-type scale.

Work connectivity behavior after-hours was measured with a six-item scale developed by Fenner and Renn (2010), which included two reverse scored items: “I leave my cell phone or WeChat turned off and do not use my computer for work-related tasks when I return home from work at night” and “I ignore job-related tasks at home at night or on weekends using my cell phone, WeChat or computer.” This scale was used to measure the behavior of employees who perform work during non-working hours. The Cronbach’s alpha for this measure was 0.906.

Work–family enrichment (WFE) was measured using a nine-item scale developed by Carlson et al. (2006). An example of an item is, “My involvement in my work helps me to understand different viewpoints and this helps me be a better family member.” The Cronbach’s alpha for this measure was 0.916.

Work–family conflict (WFC) was measured with a nine-item scale developed by Carlson et al. (2000). An example of item wording is, “My work keeps me from my family activities more than I would like.” The Cronbach’s alpha for this measure was 0.917.

Thriving at work (TW) was measured with a 10-item scale developed by Porath et al. (2012). An example item is, “At work, I find myself learning often.” The Cronbach’s alpha for this measure was 0.857.

Thriving at family (TF) was measured with a 10-item scale developed by Porath et al. (2012). We changed work content to family content. An example item is, “At family, I find myself learning often.” The Cronbach’s alpha for this measure was 0.847.

Family member support (WS) was measured with a seven-item scale developed by Boyar et al. (2014). An example item is, “My family is willing to listen to me when I talk about work.” The Cronbach’s alpha for this measure was 0.763.

To reduce the influence of demographic characteristics and family environment on the results, we controlled for factors such as participants’ age, gender, education, position, marital status, number of children, and the office environment in the home (Higgins et al., 1994; Minnotte et al., 2010). Females were coded as 0, males were coded as 1. Age 18–30 years was coded as 1, 31–40 as 2, 41–50 as 3, and 51–60 as 4. An education level of high school or below was coded as 1, 3-year college diploma as 2, bachelor degree as 3, and master degree as 4. Ordinary employees were coded as 1, front-line managers as 2, middle managers as 3, and senior managers as 4. Unmarried participants were coded as 0, and married ones as 1. People with no more than one child were coded as 0, and those with more than one child as 1. In regard to parents help, those who received none were coded as 1, those who rarely received help as 2, those with occasional help as 3, and those who often received help as 4. An enclosed independent office space was coded as 1, a non-enclosed independent office space as 2, and an open office space as 3.

## Analysis and Results

### Confirmatory Factor Analysis

To check whether WCBA, work–family conflict, work–family enrichment, thriving at work, thriving at family, and family member support could be mutually discriminated, we used Mplus7.4 to conduct confirmatory factor analysis (CFA). We compared the six-factor model with a one-factor model, and other factor models. The results, presented in Table 1, show that the six-factor model (χ^2^/df = 2.117, CFI = 0.816, TLI = 0.806, RMSEA = 0.066, SRMR = 0.068) was better than any other alternative construct model. The CFA results also indicate that the respondents could distinguish all the constructs clearly.

**TABLE 1:** Results for confirmatory factor analysis.

Model	χ^2^	df	χ^2^/df	CFI	TLI	RMSEA	SRMR
One factor	5544.584	1224	4.530	0.413	0.388	0.117	0.138
Two factors	4395.757	1223	3.594	0.569	0.550	0.100	0.117
Three factors	3829.163	1221	3.136	0.645	0.630	0.091	0.100
Four factors	3404.161	1218	2.795	0.703	0.689	0.084	0.092
Five factors	2885.524	1214	2.377	0.773	0.761	0.073	0.074
Six factors	2559.457	1209	2.117	0.816	0.806	0.066	0.068

*One factor: WCBA + WFC + WFE + TF + TW + WS.*

*Two factors: WFC − WCBA + WFE + TF + TW + WS.*

*Three factors: WFC − WCBA + WFE + TW + WS − TF.*

*Four factors: WCBA − WFC − WFE − TW + TF + WS.*

*Five factors: WCBA − WFC − WFE − TF − TW + WS.*

*Six factors: WCBA − WFC − WFE − TW − TF − WS.*

### Descriptive Analysis

We also checked the common methods bias and the means, standard deviations, and correlations among the demographic and six core research variables, using SPSS21.0. The variance explained by the first factor was 24.8%, which is lower than 50% of the total explanatory variance of 67.7%, indicating lack of any serious common method bias problem in this study. The descriptive analysis of the study variables resulted in means (standard deviations) for WCBA, work–family enrichment, work–family conflict, thriving at work, thriving at family, and family member support, of 3.98 (0.79), 4.18 (0.57), 2.78 (0.95), 4.25 (0.45), 4.07 (0.55), and 4.15 (0.51), respectively. There was a significant positive correlation between WCBA and work–family enrichment (*r* = 0.21, *p* < 0.01), work–family conflict (*r* = 0.18, *p* < 0.01), and thriving at work (*r* = 0.28, *p* < 0.01), but not with thriving at family (*r* = 0.07, *p* > 0.05). Work–family enrichment was positively related to thriving at work (*r* = 0.80, *p* < 0.01), thriving at family (*r* = 0.47, *p* < 0.01), and family member support (*r* = 0.47, *p* < 0.01). Work–family conflict was negatively related to thriving at work (*r* = −0.17, *p* < 0.01), thriving at family (*r* = −0.46, *p* < 0.01) and family member support (*r* = −0.15, *p* < 0.05), which provided a preliminary test of the study hypothesis.

### Hypothesis Testing

We used hierarchical regression and bootstrapping in SPSS21.0 to test the research hypotheses, leading to the results shown in Table 2. Models 1, 3, 5, and 9 include the regression results for the control variables work–family conflict, work–family enrichment, thriving at family, and thriving at work, respectively. Models 2, 4, 6, and 10 include the regression results for WCBA to work–family conflict, work–family enrichment, thriving at family, and thriving at work, respectively. The results show that WCBA had a positive effect on work–family conflict (β = 0.205, *p* < 0.01), work–family enrichment (β = 0.158, *p* < 0.01), and thriving at work (β = 0.244, *p* < 0.01), but no significant effect on thriving at family (β = 0.030, *p* > 0.05), supporting H1a, but not H1b. Model 8 shows that WCBA was not significantly related to thriving at family, but work–family enrichment was significantly related to thriving at family. Model 12 shows that WCBA and work–family enrichment were significantly related to thriving at work. Model 4 shows that WCBA was significantly related to work–family enrichment. Combining Models 8 and 12 with Model shows that work–family enrichment played a mediating role in the relationship between WCBA and thriving at family (indirect effect = 0.050, 95% confidence interval [CI] = [0.010, 0.097]) and thriving at work (indirect effect = 0.070, 95% CI = [0.015, 0.135]). Among them, model 4,8,12 played a fully mediating role in the thriving of the family; thus both H2a and H2b were supported. Model 7 shows that WCBA and work–family conflict were significantly related to thriving at family. Model 11 shows that WCBA and work–family conflict were significantly related to thriving at work. As before, combining Models 7 and 11 combined with Model 2 shows that work–family conflict played a mediating role in WCBA and thriving at family (indirect effect = −0.069, 95% CI = [−0.117, −0.028]) and thriving at work (indirect effect = −0.023, 95% CI = [−0.044, −0.007]); thus H3a and H3b were supported.

**TABLE 2:** Results for regression analysis.

Model	1	2	3	4	5	6	7	8	9	10	11	12
				
Variables	WFC		WFE		TF				TW			
Gender	0.020	0.016	–0.012	–0.016	–0.046	–0.046	–0.038	–0.039	–0.016	–0.021	–0.018	–0.009
Age	−0.172*	−0.167*	–0.074	–0.070	–0.029	–0.029	–0.110	0.003	–0.101	–0.095	−0.128*	–0.041
Education	0.087	0.070	–0.088	–0.101	–0.033	–0.036	–0.001	0.010	–0.092	–0.112	–0.098	–0.035
Position	–0.056	–0.085	0.277***	0.255***	0.118	0.114	0.072	–0.003	0.232**	0.198**	0.181**	0.002
Marital status	0.117	0.080	0.124	0.096	0.142	0.137	0.176*	0.093	0.123	0.080	0.096	0.006
Child	–0.147	–0.128	0.005	0.020	–0.090	–0.088	–0.150	–0.097	–0.033	–0.010	–0.035	–0.025
Parents help	–0.107	–0.107	0.204**	0.203**	0.147*	0.147*	0.094	0.054	0.141*	0.140*	0.119*	–0.016
Home work space	0.095	0.107	–0.074	–0.065	–0.108	–0.107	–0.054	–0.077	−0.143*	−0.129*	–0.108	−0.079*
WCBA		0.205**		0.158**		0.030	0.130*	–0.042		0.244***	0.284***	0.123**
WFC							−0.490***				−0.198**	
WFE								0.457***				0.766***
*R*	0.073	0.112	0.139	0.163	0.063	0.064	0.277	0.239	0.108	0.164	0.198	0.656
*F*	2.432	3.468	5.013	5.328	2.087	1.874	9.426	7.725	3.756	5.376	6.091	46.862
*N*	257	257	257	257	257	257	257	257	257	257	257	257

***p < 0.001, **p < 0.01, *p < 0.05.

We analyzed the interactive effects of work–family enrichment and work–family conflict with family member support using PROCESS. The results indicate that the interaction between work–family enrichment and family member support was significantly related to thriving at work (β = 0.212, 95% CI = [0.112, 0.311]), and that work–family enrichment had a weaker effect on thriving at work at a lower level of family member support (β = 0.543, 95% CI = [0.467, 0.620]). Further, work–family enrichment had a stronger effect on thriving at work at a higher level of family member support (β = 0.760, 95% CI = [0.662, 0.858]); thus H4a was supported. The interaction between work–family enrichment and family member support also significantly positively affected thriving at family (β = 0.219, 95% CI = [0.046, 0.391]); work–family enrichment had a weaker effect on thriving at family at lower levels of family member support (β = 0.247, 95% CI = [0.114, 0.380]); and work–family enrichment had a stronger effect on thriving at family at higher levels of family member support (β = 0.471, 95% CI = [0.301, 0.641]). Thus, H4b was supported. The interaction between work–family conflict and family member support significantly negatively affected thriving at work (β = −0.143, 95% CI = [−0.246, −0.039]); at low levels of family member support (when support level was less than 2.84), the inhibitory effect of work–family conflict on thriving at work was enhanced (β = 0.153, 95% CI = [0.000, 0.305]); and at higher levels of family member support, the inhibitory effect of work–family conflict on thriving at work was weakened (β = −0.109, 95% CI = [−0.180, −0.037]), so H4c was supported. However, the interaction between work–family conflict and family member support did not have a significant effect on thriving at family (β = −0.033, 95% CI = [−0.148, 0.081]), so H4d was not supported.

**FIGURE 2:**
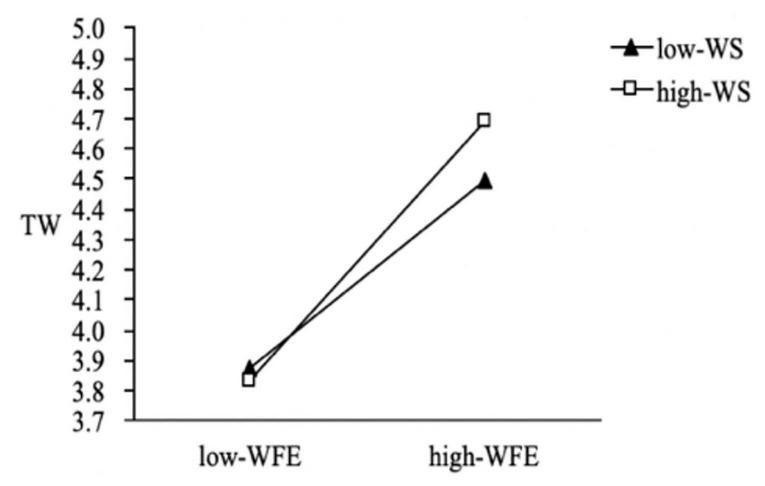
The moderating effect of family member support on the influence of WFE on TW.

**FIGURE 3:**
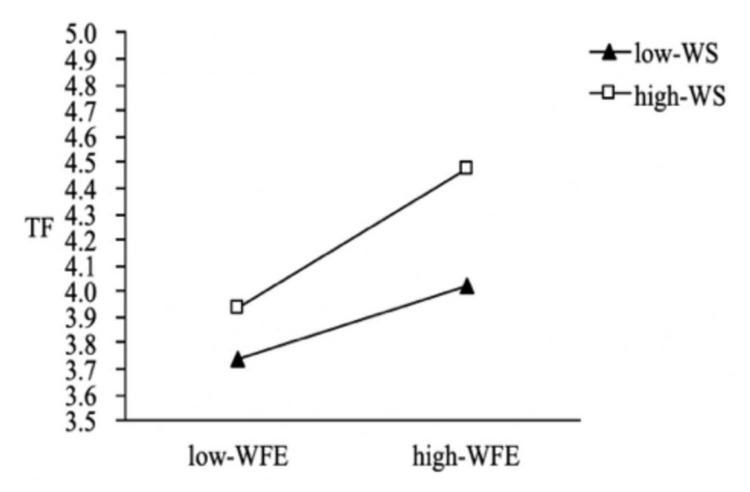
The moderating effect of family member support on the influence of WFE on TF.

**FIGURE 4:**
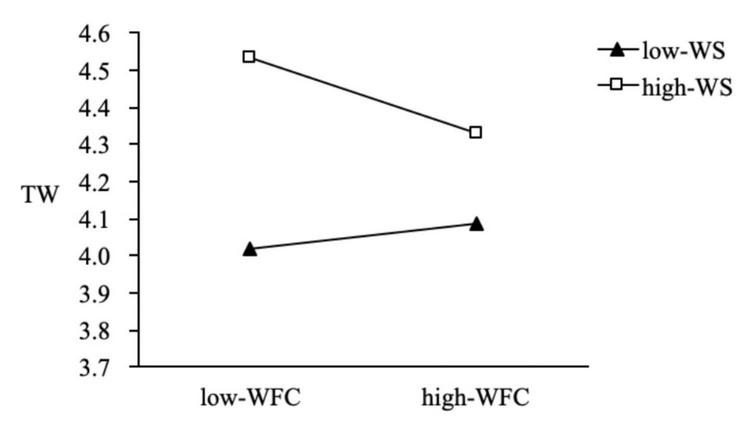
The moderating effect of family member support on the influence of WFC on TW.

We also used PROCESS macros to examine the whole moderated mediation model. The results indicate that the indirect effect of work–family enrichment on thriving at work (β = 0.06, 95% CI = [0.013, 0.119]) and thriving at family (β = 0.02, 95% CI = [0.005, 0.061]) was weakened with a lower level of family member support. In contrast, the indirect effect of work–family enrichment on thriving at work (β = 0.084, 95% CI = [0.018, 0.162]) and thriving at family (β = 0.057, 95% CI = [0.011, 0.118]) was stronger with a higher level of family member support; thus H5a and H5b were supported (Figures 2, 3). The results also indicate that the indirect effect of work–family conflict (β = 0.002, 95% CI = [−0.030, 0.028]) on thriving at work was not significant at the lower family member support level, but the indirect effect (β = −0.031, 95% CI = [−0.057, −0.009]) weakened the negative effect of work–family conflict at a higher level of family member support (Figure 4); thus H5c was supported. Regardless of the level of family member support, it did not affect the indirect effect of work–family conflict on thriving at family; thus H5d was not supported.

**FIGURE 5:**
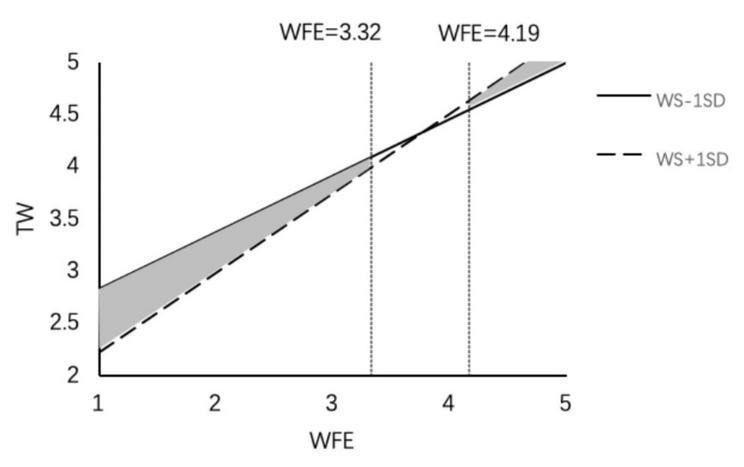
The moderating effect of family member support on the influence of WFE on TW.

**FIGURE 6:**
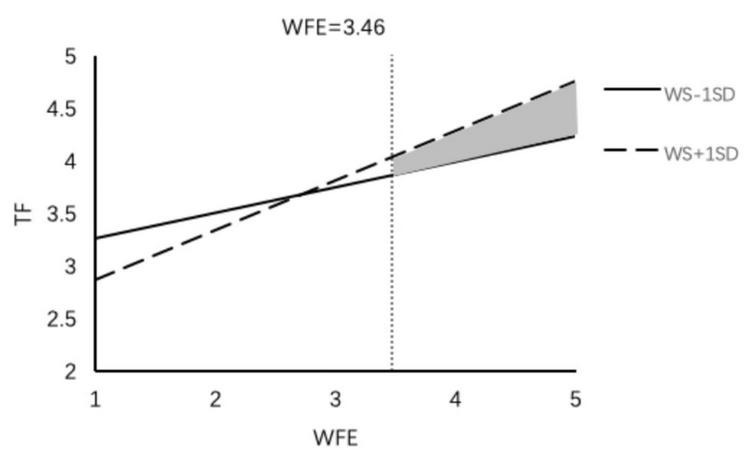
The moderating effect of family member support on the influence of WFE on TF.

**FIGURE 7:**
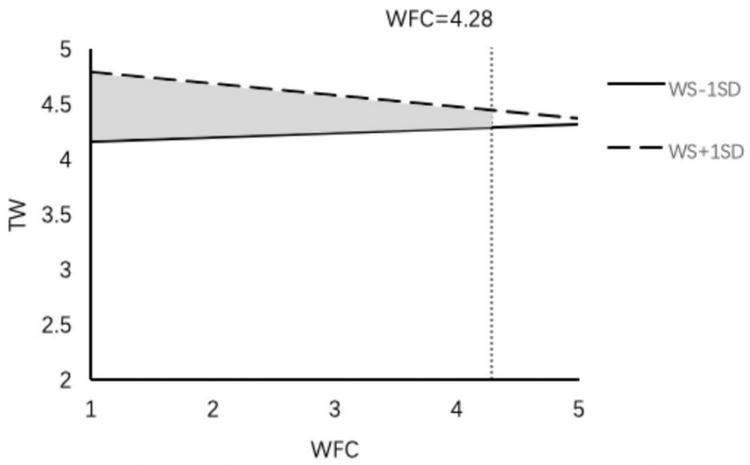
The moderating effect of family member support on the influence of WFC on TW.

**FIGURE 8:**
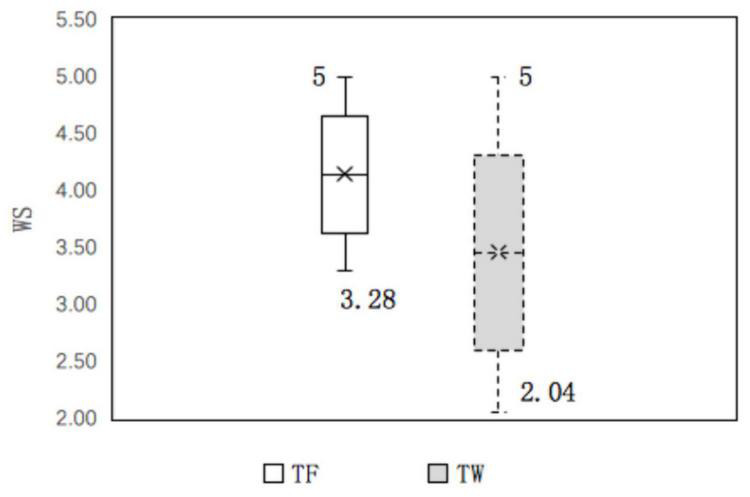
The different moderating effect of family member support between TF and TW.

We used the J-N approach to explore the conditions under which family members supported a boundary effect (Spiller et al., 2012). The results indicate that family member support played a different role at different levels of work–family enrichment and work–family conflict. (1) In the work–family enrichment path, family member support had a significant positive impact on thriving at work (Figure 5). When work–family enrichment was less than 3.32 points, lower family member support strengthened the positive effect of work–family enrichment on thriving at work, and when work–family enrichment was higher than 4.19 points, higher family member support strengthened the positive effect of work–family enrichment. At the same time, family member support had a significantly positive effect on thriving at family (Figure 6). When work–family enrichment was higher than 3.46 points, higher family member support strengthened the positive impact of work–family enrichment on thriving at family. (2) In the work–family conflict path, family member support had a significantly negative impact on thriving at work (Figure 7). When the value for work–family conflict fell below 4.28 points, higher family member support weakened the negative impact of work–family conflict on thriving at work. (3) For the work–family enrichment pathway, the different degree of family member support varied significantly between thriving at work and thriving at family (Figure 8). When family member support had a value higher than 2.04 points, the positive impact of work–family enrichment on thriving at work was strengthened, and when family member support had a value above 3.28 points, the impact on thriving at family was strengthened. The means for work–family enrichment and work–family conflict in this study were 4.16 points and 2.78 points, so the J–N method provided further support for the moderating effects in this study.

## Discussion

### Discuss of Results

Our analysis of the double-edged sword of the effect between WCBA and thriving at work and thriving at family of employees led to several conclusions. First, WCBA can promote the thriving at work of employees (H1a), but does not have a significant effect on thriving at family (H1b). Work–family enrichment plays a positive mediating role between WCBA and thriving at work (H2a) and thriving at family (H2b). Work–family conflict plays a negative mediating role between WCBA and thriving at work (H3a) and thriving at family (H3b). Family member support plays a positive moderating role between work–family enrichment and thriving at work (H4a) and thriving at family (H4b), and positively moderated the indirect effects of work–family enrichment (H5a, H5b). Family member support plays a negative moderating role between work–family conflict and thriving at work (H4c). It negatively moderates the indirect effects of work–family conflict (H5c), but cannot moderate between work–family conflict and thriving at family (H4d) and the indirect effects of work–family conflict (H5d).

Work connectivity behavior after-hours can promote employees’ thriving at work, but cannot directly promote thriving at family. Studies have shown that during the COVID-19 epidemic, working from home for a long time enhanced employees’ loneliness, triggered social isolation, inhibited employees’ need for face-to-face communication, and caused work anxiety, resulting in negative work conditions for employees. WCBA can enhance the connection between employees and the organization, effectively alleviate the isolation of working from home, and meet the communication and feedback needs of employees during non-working hours. Further, not only can it alleviate the negative states of employees, but can even promote thriving at work. However, WCBA is an extension of the passive work adopted by employees at home, which encroaches on their non-working hours to ensure that work tasks can be completed smoothly, and encroaches on the family fields of employees; thus, it is difficult to directly have a positive impact on thriving at family.

Further research identified a double-edged sword effect between WCBA and thriving at work and family, in that WCBA can have different effects on thriving at work and family through work–family enrichment and work–family conflict. WCBA can enhance employees’ abilities to switch roles, enabling employees to better balance work and family roles to achieve work–family enrichment. Further, the status of work–family enrichment can improve the responsiveness and efficiency of employees working from home, promoting employees’ thriving at work. However, WCBA also increases the demand for work during non-working hours, resulting in conflict between the needs of work and family, which interrupts the continuity and stability of work, and inhibits the thriving at work of employees. Conversely, WCBA can expand employees’ abilities to access resources, and facilitate the flow of surplus work resources to the household sector to achieve work–family enrichment, promoting employees’ thriving at family. However, WCBA can also interrupt the continuity of employees’ family roles, deplete employees’ resources for performing family duties, create work–family conflict, and inhibit thriving at family.

The support of family members is an important boundary condition for the double-edged sword effect between WCBA and thriving at work and family, but the boundary effect of family member support is not always present and stable. In thriving at work and family, different levels of family member support have different effects. While family member support plays a moderating role in work–family enrichment and thriving at work and family, the moderating role of thriving at work is more sensitive than that of thriving at family (e.g., Figure 8). Family member support (2.04 points) was more likely to influence the effect of work–family enrichment on thriving at work, while an impact on thriving at family required a higher level of family member support (3.28 points). This suggests that in a tough employment environment, employees are motivated to prioritize the resources that they receive for job roles to reduce the risk of unemployment.

There are differences in the boundary effects that different levels of support of family members produce in work–family enrichment and work–family conflict. First, the support of family members can reinforce work–family enrichment by promoting thriving at work and the indirect effects of work–family enrichment. This shows that a higher level of family member support can reduce expectations of the family roles assumed by employees, create a good working and family environment for employees to continue to devote themselves to work roles, and help employees achieve thriving at work. However, under different levels of work–family enrichment, the support of family members has different positive effects on work–family enrichment and thriving at work. Compared with lower family member support levels, higher levels weakened the positive effect of work–family enrichment (less than 3.32 points) on thriving at work, because higher family member support may have increased the psychological burden of employees; higher family member support strengthened the positive impact of work–family enrichment (higher than 4.19 points) on thriving at work, because employees regarded family support as a resource to strengthen the positive impact of work–family enrichment on thriving at work; family member support had no weakening or strengthening effect on the positive effect on work–family enrichment (between 3.32 and 4.19 points) and thriving at work, because employees were more likely to regard family member support as a resource to promote work–family enrichment. This suggests that for different states of work–family enrichment, employees expect to perceive different levels of support of family members.

Second, the support of family members can weaken the inhibiting and indirect effects of work–family conflict on thriving at work. This shows that a higher level of family member support can make up for the vacancy of family roles caused by employees due to work, effectively reduce family interference with work, and fully improve the threshold of work–family conflict, which is conducive to the realization of thriving at work. However, the weakening effect of family member support is not always present. Higher family member support weakened the inhibitory effect of work–family conflicts (below 4.28 points) on thriving at work compared to lower family support. This suggests that family member support can only play a role when work–family conflict is contained within a certain range, alleviating the inhibitory effect of work–family conflict on thriving at work. Conversely, when work–family conflict is too great, the support of family members will have no effect.

Third, the support of family members can strengthen the promotion and indirect effect of work–family enrichment on thriving at family, indicating that higher family member support can enhance the willingness of employees to switch from work roles to family roles, promote employees to be more willing to perform family roles, and realize their thriving at family. However, the support of family members needs to be based on a certain amount of work–family enrichment to be able to play a role. Higher family member support promoted the transformation of work–family enrichment (higher than 3.46 points) to thriving at family compared with lower family member support. This suggests that in terms of thriving at family, the boundary effect of family support needs to be based on a certain level of work–family enrichment.

Finally, the support of family members cannot influence the inhibitory effect of work–family conflict on thriving at family. Because work–family conflict prevents effective support for employees, and employees will avoid work–family conflict by increasing thriving at work, they cannot perceive support from family members after leaving the family environment.

### Theoretical Contributions

The application of role theory in the field of work and family has been expanded from the perspective of dynamic transformation. Based on the perspective of dynamic role shifting, we reveal the double-edged sword impact of WCBA on both thriving at work and thriving at family. WCBA increases the frequency and intensity of employee role transitions, and role theory studies have focused on the conflict and transition between multiple roles and their impact on work and family. However, our study focused on how the continuous, dynamic shift between multiple roles of work and family affects thriving at work and thriving at family during non-working hours. The double-edged sword effect of thriving at work was analyzed from the perspective of role flexibility, and the double-edged sword effect of thriving at family was analyzed from the perspective of role permeability. Our research enriches role theory in the field of work and family for dynamic and continuous role transformation.

The scenario boundaries of WCBA has been expanded from the perspective of passive adaptation, the positive effect of WCBA on thriving at work has been supplemented, and the double-edged sword impact of WCBA on thriving at work revealed. WCBA extends employees’ working time and space; previous studies have focused on the passive connectivity of employees in maintaining work–family boundaries, and the impact on work stress, work–family conflict, and work performance. In the context of telecommuting exacerbating the blurring of work–family boundaries, we focused on how WCBA affects employee thriving at work in the process of employees’ adaptation to boundary maintenance and boundary blurring. From the perspective of role flexibility, this shows that WCBA leads to work–family enrichment and work–family conflict, and has a double-edged sword effect on promoting and inhibiting thriving at work. This has expanded the contextual boundaries of WCBA affecting employees’ working status, and constructed a path for WCBA to have a positive impact on thriving at work.

We expand on the factors that influence the thriving at family from the perspective of WCBA; the positive effect of WCBA on thriving at family has been supplemented; and the double-edged sword impact of WCBA on thriving at family has been revealed. WCBA weakens original family boundaries; previous studies have focused on the negative impact of the role and resource competition arising from non-working-time work connectivity on thriving at family. We focused on how the mutual flow of work–family resources that forms in the process of interpenetration and blurring of work–family boundaries affects the thriving at family of employees. From the perspective of role permeability, this shows that WCBA leads to work–family enrichment and work–family conflict, and has a double-edged sword effect on promoting and inhibiting thriving at family. In this way, the role of WCBA has been expanded from the work field to the family field, and its positive impact on thriving at family has been constructed.

The boundary effect of family member support on the double-edged sword of thriving at work and thriving at family have been revealed in the family field, and use of the J–N method has revealed the impact of family member support on thriving at work and thriving at family under different levels of work–family enrichment and work–family conflict. As the main place where WCBA occurs in the family field, the boundary effect of WCBA has been studied in previous studies, mainly from the perspective of individual demand satisfaction, role integration preferences, and organizational norms. We focused on the sensitivity of family members to the penetration of work into the boundaries of the family. From the perspective of family members, pointing out higher family member support can help reduce the sensitivity of blurred boundaries. Understanding of the relationship between work connectivity after-hours and the work and family spheres has been deepened. At the same time, we analyzed the boundary effect of different levels of family member support on work–family enrichment and work–family conflict, deepening understanding of when and how family member support plays a boundary role, and more comprehensively explaining the impact of the boundary between WCBA and the work and family fields.

### Practical Implications

Organizations need to expand the facilitating effect of work–family enrichment and reduce the inhibitory effect of work–family conflict when adopting WCBA. Studies have shown that work–family enrichment and work–family conflict can have a double-edged sword effect on thriving at work and family. Particularly during the COVID-19 epidemic, employees have passively taken on remote work for a long time, which has highlighted the impact of work–family enrichment and conflict on the work and family fields. Thus, in the process of adopting WCBA, organizations need to pay attention to the sensitivity of the different family needs of employees to connected work, and must take targeted measures to strengthen the work–family enrichment of employees and alleviate work–family conflict. For example: Distribute work tasks appropriately. According to the family environment of different employees, the adverse influence of connected work on work and family can be reduced by rationally allocating work and giving employees more autonomy to adjust. At the same time, clarify the content and boundary of WCBA. When using WCBA, organizations should clarify the content and boundary of work in advance to reduce redundancy, improve efficiency. Build a clean, fast and effective connection mechanism, reduce the encroachment of non-working-time-connected work on the family lives of employees, and reserve more energy for employees to balance their work and family.

Organizations could provide benefits for family members to support employees, which will influence work–family enrichment and conflict, contributing to work and family prosperity in both positive and negative respects. organizations can employ measures such as health insurance, child and parent care, family care, and family travel for family members, to go some way toward make up for the missing family roles of employees, and encourage family members to form a higher level of support for employees’ work. Further, organizations can take on timely and visible positive feedback regarding WCBA to better understand employee motivations and family needs for higher family member support; for example, by providing career growth opportunities such as promotion and training, and establishing special mechanisms of reward and punishment.

Because the boundary role of family member support needs to be effective at a certain level of work–family enrichment and conflict, organizations need to take targeted measures to fully utilize the support of family members. Organizations need to grasp the level of work–family enrichment of employees in WCBA, to improve the degree of work–family enrichment by improving feedback regarding WCBA, promoting the career growth of employees, and increasing family benefits. Further, organizations need to understand the level of work–family conflict among employees, which may reduce the degree of work–family conflict by improving the efficiency of WCBA, reducing the frequency of connectivity, and standardizing connectivity systems.

## Conclusion

This study found how WCBA affects employees’ work and family prosperity. WCBA can promote the thriving at work of employees, but does not have a significant effect on thriving at family. There is a double-edged sword effect between WCBA and thriving at work and family, in that WCBA can have different effects on thriving at work and family through work–family enrichment and work–family conflict. The support of family members is an important boundary condition for the double-edged sword effect between WCBA and thriving at work and family, but the boundary effect of family member support is not always present and stable, there are differences in the boundary effects that different levels of support of family members produce in work–family enrichment and work–family conflict. The study has contributed to understanding the relationship between WCBA and thriving at work and family, which also makes organizations to better help employees’ thriving at work and family in the WCBA.

## Limitations and Future Research

This research has the following shortcomings in its research process, which need to be improved in future research. First, the theoretical model was mainly tested using cross-sectional data, and lack of revealed the dynamic process by which WCBA affects thriving at work and family. A longitudinal data collection method could be considered in subsequent studies to more fully reflect the complex relationship between work connectivity, and work and family prosperity. Second, While the positive influence of WCBA on thriving at work and family have been focused, the negative influence of WCBA on thriving at work and family have been ignored, in future studies, the negative influence of WCBA on thriving at work and family will be focused. Third, although we combined the work and family fields for analysis, we still measured family member support in the form of employee reporting. As we did not measure the actual perception of family members, a follow-up study will analyze this at different levels to more fully understand the complex relationship between WCBA and work and family thriving. Finally, we analyzed the boundary effect from the family field, but the organizational scenario is also an important boundary condition affecting this mechanism; thus, seeking to understand the boundary effect of the family scenario and the organizational scenario on this influence mechanism in a follow-up study will help us grasp the integrity of the impact of WCBA on work and family prosperity.

## Data Availability Statement

The raw data supporting the conclusions of this article will be made available by the authors, without undue reservation.

## Ethics Statement

The studies involving human participants were reviewed and approved by Ethics Review Committee of the School of Management, Harbin Institute of Technology. Written informed consent from the participants’ legal guardian/next of kin was not required to participate in this study in accordance with the national legislation and the institutional requirements.

## Author Contributions

YY, RY, and YM took part in this study. YY and YM mainly conceived the idea and basic model of this study. RY collected and analyzed data and wrote the manuscript. All authors have read and agreed to the published version of the manuscript.

## Conflict of Interest

The authors declare that the research was conducted in the absence of any commercial or financial relationships that could be construed as a potential conflict of interest.

## Publisher’s Note

All claims expressed in this article are solely those of the authors and do not necessarily represent those of their affiliated organizations, or those of the publisher, the editors and the reviewers. Any product that may be evaluated in this article, or claim that may be made by its manufacturer, is not guaranteed or endorsed by the publisher.
